# Comprehensive Characterization of Serum MicroRNA Profile in Response to the Emerging Avian Influenza A (H7N9) Virus Infection in Humans

**DOI:** 10.3390/v6041525

**Published:** 2014-04-02

**Authors:** Zheng Zhu, Yuhua Qi, Aihua Ge, Yefei Zhu, Ke Xu, Hong Ji, Zhiyang Shi, Lunbiao Cui, Minghao Zhou

**Affiliations:** 1 Institute of Pathogenic Microbiology, Jiangsu Provincial Center for Disease Control and Prevention, Key Laboratory of Enteric Pathogenic Microbiology, Ministry of Health, Nanjing 210009, China; E-Mails: zhengzhu@jscdc.cn (Z.Z.); qiyuhua@jscdc.cn (Y.Q.); shizhiyang@jscdc.cn (Z.S.); 2 Department of Acute Infectious Diseases Control and Prevention, Jiangsu Provincial Center for Disease Control and Prevention, Nanjing 210009, China; E-Mails: geah@jscdc.cn (A.G.); jszyf@jscdc.cn (Y.Z.); jsxk@jscdc.cn (K.X.); jih@jscdc.cn (H.J.)

**Keywords:** H7N9 virus, avian influenza, serum microRNA, expression profile

## Abstract

A novel avian-origin influenza A (H7N9) virus recently occurred in China and caused 137 human infection cases with a 32.8% mortality rate. Although various detection procedures have been developed, the pathogenesis of this emerging virus in humans remains largely unknown. In this study, we characterized serum microRNA (miRNA) profile in response to H7N9 virus infection using TaqMan Low Density Arrays. Upon infection, a total of 395 miRNAs were expressed in the serum pool of patients, far beyond the 221 in healthy controls. Among the 187 commonly expressed miRNAs, 146 were up-regulated and only 7 down-regulated in patients. Further analysis by quantitative RT-PCR revealed that the serum levels of miR-17, miR-20a, miR-106a and miR-376c were significantly elevated in patients compared with healthy individuals (*p* *<* 0.05). Receiver operating characteristic (ROC) curves were constructed to show that each miRNA could discriminate H7N9 patients from controls with area under the curve (AUC) values ranging from 0.622 to 0.898, whereas a combination of miR-17, miR-20a, miR-106a and miR-376c obtained a higher discriminating ability with an AUC value of 0.96. Our findings unravel the significant alterations in serum miRNA expression following virus infection and manifest great potential of circulating miRNAs for the diagnosis of viral diseases.

## 1. Introduction

A novel avian-origin influenza A H7N9 virus recently emerged in eastern China and was associated with three fatal human cases [1]. Although genomic sequences of the H7N9 virus were identified by sequencing based on the virus isolates, the possible source of infections and the reservoirs of this reassortant virus are still unclear. Patients with H7N9 virus infection generally presented severe clinical symptoms characterized by rapidly progressive pneumonia, acute respiratory distress syndrome (ARDS) and multiorgan failure [1,2,3]. As of 25 October 2013, a total of 137 laboratory-confirmed human infection cases, including 45 deaths, have been reported [4], thereby highlighting the great threat to human beings. Since the emerging H7N9 virus is a novel reassortant influenza virus and has not been detected previously in humans or animals, little is known about the molecular mechanisms responsible for virus infection. Understanding the viral pathogenesis might allow the development of prophylactic and therapeutic strategies.

MicroRNAs (miRNAs) represent a class of ~22 nucleotide (nt) non-coding RNA molecules that negatively regulate gene expression at the post-transcriptional level, either by the cleavage of mRNAs or by the repression of protein translation depending on the degree of sequence complementarity between miRNAs and their target mRNAs [5]. Bioinformatic prediction indicates that over 30% of all protein-coding genes may be regulated by miRNAs in mammals [6], emphasizing the potential significance of miRNAs as major post-transcriptional regulators. These endogenous small RNAs have already been shown to play important roles in diverse biological events, such as development, proliferation, differentiation, apoptosis and tumor development [7,8]. miRNAs were initially reported to be present in body fluids like serum samples from humans and various animals in 2008 [9,10]. Intensive studies were subsequently performed to reveal altered serum miRNA expression profiles involved in a variety of pathological conditions [11,12,13,14]. Circulating miRNAs are usually packaged into exosome-like microparticles that can protect them against endogenous RNase digestion, and are therefore highly stable in body fluids [15]. This unique advantage has made them as ideal and powerful biomarkers for the diagnosis of Alzheimer’s disease [16], diabetes [17], cardiovascular disease [18] and cancers [19,20,21]. Similarly, these cell-free small RNAs are also applied to potent detection of infectious diseases. Altered serum miRNA expression profiles were noted in patients with hand, foot and mouth disease (HFMD), caused mainly by enterovirus 71 (EV71) and coxsackievirus A16 (CVA16). In addition, six miRNAs (miR-148a, miR-143, miR-324-3p, miR-628-3p, miR-140-5p and miR-362-3p) possessed the ability to discriminate patients with enterovirus infections from healthy controls [22]. Another study by Shrivastava *et al.* revealed that the plasma/serum levels of miR-20a and miR-92a in HCV-infected patients were significantly increased when compared with healthy volunteers, highlighting the clinical potential of these two miRNAs as sensitive biomarkers for early detection of HCV infection [23].

In this study, we globally analyzed the serum miRNA expression profile in response to H7N9 virus infection in humans and further evaluated the diagnostic potential of circulating miRNA candidates.

## 2. Results and Discussion

### 2.1. Sample Characteristics

During the outbreak of H7N9 virus infection, a total of 57 participants were enrolled in our study, including 36 healthy controls (19 men and 17 women, mean age: 48.6 ± 11.6) and 21 patients (13 men and 8 women, mean age: 51.9 ± 18.7), of which 10 participants from each group were selected for the TaqMan Low Density Array assays. The detailed information about research subjects are listed in Table 1. There are no statistical differences in age and sex distribution between healthy controls and H7N9 virus-infected patients.

**Table 1:** The basic characteristics of healthy controls and patients with H7N9 virus infection.

Sample Characteristic	TaqMan Low-Density Array		Validation Study	
	Controls	Patients	Controls	Patients
Number	10	10	36	21
Sex (Male/Female)	6/4	6/4	19/17	13/8
Age (Years, mean ± SD)	52.1 ± 17.2	54.0 ± 19.4	48.6 ± 11.6	51.9 ± 18.7

### 2.2. Global Analysis of Serum MiRNA Expression Profiles

**Figure 1:**
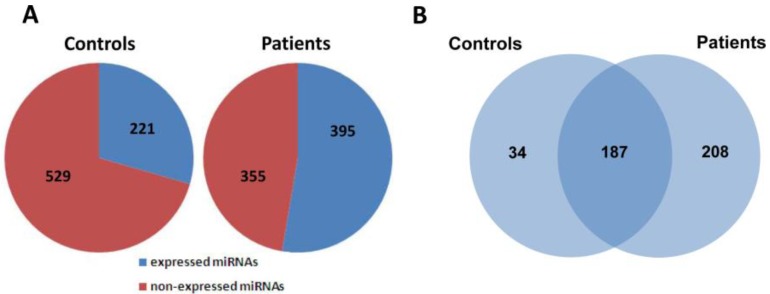
Global analysis of serum miRNA expression profiles between healthy controls and H7N9 virus-infected patients. (**A**) The distribution of expressed and non-expressed miRNAs in serum pools of controls and patients; (**B**) 187 miRNAs were commonly expressed in both groups, whereas 34 and 208 miRNAs were expressed in serum pools of controls and patients, respectively.

To investigate the possible changes of serum miRNA expression in response to H7N9 virus infection, we performed miRNA profile examination based on the TaqMan Low Density Array Human MiRNA Panel A + B which could simultaneously detect the expression levels of 750 miRNA molecules. Upon virus infection, 395 miRNAs were found to be expressed in the serum pool of H7N9 patients, whereas only 221 out of 750 miRNAs were detected in that of healthy controls (Figure 1A). Further analysis revealed that 187 miRNAs were commonly detected in both research groups with the remaining 34 and 208 molecules exclusively expressed in controls and patients, respectively (Figure 1B). In order to identify miRNAs tightly associated with H7N9 virus infection, differential expression of miRNAs between two groups must meet the following criteria: (1) Ct values *<*35 to ensure reliable detection; (2) ≥2-fold difference for a specific miRNA between the control and patient groups [24]. A total of 153 out of 187 commonly expressed miRNAs were found to meet these two criteria, of which 146 were up-regulated and 7 were down-regulated in the patient samples as compared with healthy controls (Appendix Table A1).

### 2.3. Confirmation of MiRNA Expression by Real Time RT-PCR

**Figure 2:**
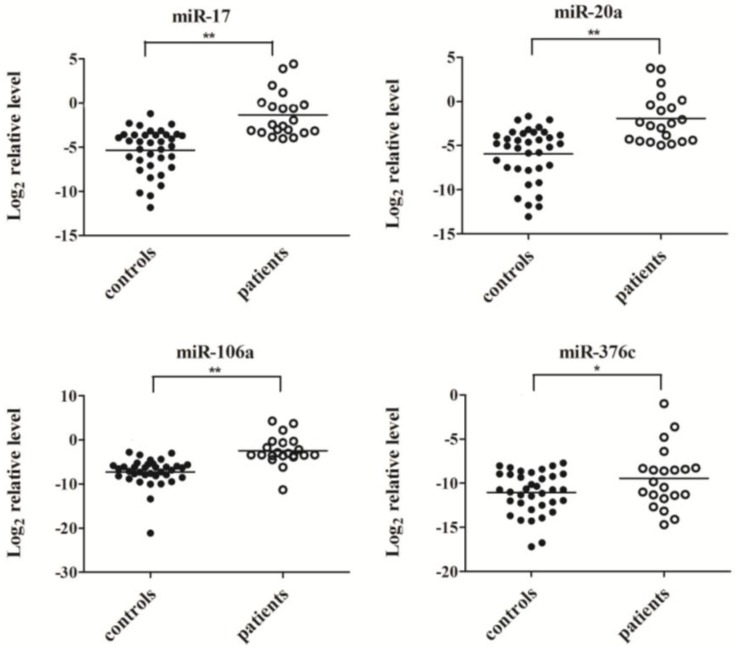
The serum levels of miR-17, miR-20a, miR-106a and miR-376c were determined by quantitative RT-PCR in individual healthy controls (*n* = 36) and patients (*n* = 21). Expression levels of four miRNAs were normalized to cel-miR-238 (Log_2_ relative level). ** *p* *<* 0.01, * *p* *<* 0.05.

Four miRNA molecules (miR-17, miR-20a, miR-106a and miR-376c) were selected for validation of the TaqMan Array data in a larger scale population by quantitative RT-PCR. As shown in Figure 2, the serum levels of miR-17, miR-20a and miR-106a were significantly elevated in response to virus infection (*p* *<* 0.01), consistent with the results from the TaqMan Arrays, while the expression of miR-376c in serum samples of H7N9 patients was slightly higher than that in healthy controls (*p* *<* 0.05).

### 2.4. Evaluation of the Diagnostic Potential of Serum miRNAs for H7N9 Virus Infection

**Figure 3:**
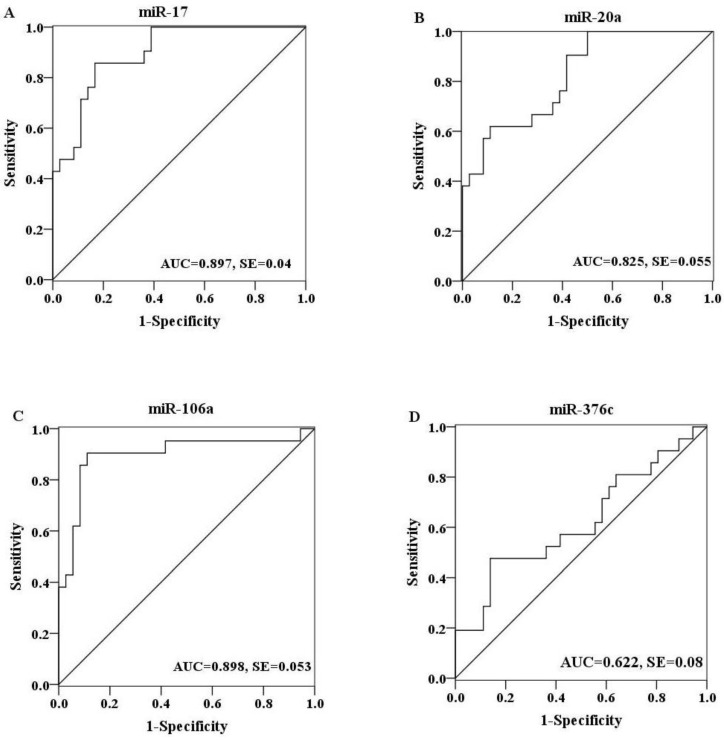
ROC curves were constructed to evaluate the diagnostic potential of serum miRNAs for H7N9 virus infection. MiR-17 (**A**), miR-20a (**B**) and miR-106a (**C**) showed a moderate discriminating efficiency with an AUC value more than 0.8, while miR-376c (**D**) exhibited a poor diagnostic ability with an AUC value of 0.622.

**Figure 4:**
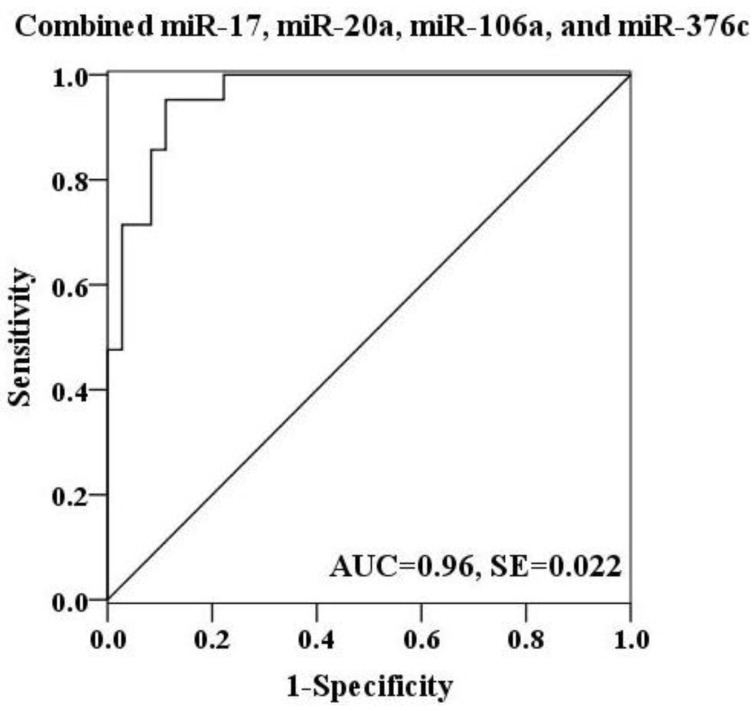
ROC curve for a combination of miR-17, miR-20a, miR-106a and miR-376c was constructed. The discriminating efficiency was apparently increased by combining above four miRNAs (AUC value: 0.96).

To further explore the diagnostic potential of these miRNAs as biomarkers for H7N9 virus infection, receiver operating characteristic (ROC) curve analysis was performed on the basis of miRNA expression levels between two groups. The ROC curves of miR-17, miR-20a and miR-106a showed a moderate distinguishing ability with a corresponding area under the curve (AUC) value of 0.897 (95% CI: 0.818–0.976), 0.825 (95% CI: 0.718–0.933) and 0.898 (95% CI: 0.798–0.998), respectively (Figure 3A–C). In contrast, the diagnostic capacity of miR-376c was relatively low for infection (AUC value: 0.622; 95% CI: 0.464–0.779) (Figure 3D). When the serum levels of these four miRNAs were subjected to combined analysis by multiple logistic regression, the generated ROC curve reflected a higher ability to differentiate patients with H7N9 virus infection from healthy controls (AUC value: 0.96; 95% CI: 0.917–1.000), demonstrating the diagnostic accuracy of miR-17, miR-20a, miR-106a and miR-376c as effective biomarkers in combination (Figure 4).

## 3. Experimental

### 3.1. Sample Collection

A total of 36 healthy volunteers and 21 H7N9 virus-infected patients including 7 fatal cases from Jiangsu Province, China were enrolled in our study. Respiratory specimens obtained from patients with influenza-like illness were first confirmed for H7N9 virus infection according to the protocol provided by the World Health Organization (WHO) [25]. Corresponding serum samples within a period of 14 days from the onset of the infection were then collected and stored at −70 °C until use. Healthy controls were recruited randomly from people who underwent a regular health check-up without clinical symptoms of any infectious disease. This study was approved by the Ethics Committee of Jiangsu Provincial Center for Disease Control and Prevention (Nanjing, China) and written informed consent was obtained from all participants.

### 3.2. RNA Extraction

Two serum pools were produced by mixing 10 samples (20 μL per sample) from each group (H7N9 patients and healthy controls) respectively for TaqMan Low Density Array assay. The pooled sera were then subjected to total RNA extraction using mirVana PARIS kit (Ambion, Austin, TX, USA) following the manufacturer’s instructions. A synthetic *Caenorhabditis elegans* miRNA (cel-miR-238 (25 fmol); Takara, Dalian, China) was added into each serum pool as an internal control prior to the isolation procedure. RNA was eluted in 100 μL of Ambion elution solution. The quality and quantity of the isolated RNA were measured in a ND-1000 NanoDrop spectrophotometer (ThermoScientific, Wilmington, DE, USA). RNA was extracted from 200 μL of individual serum sample used for real time RT-PCR assays as described above.

### 3.3. miRNA Expression Profile Examination Using TaqMan Low Density Arrays

miRNA expression profiles of two serum pools were examined using TaqMan Low Density Arrays (V3.0, Applied Biosystems, CA, USA) as described previously [26]. Briefly, 50 ng of purified total RNA was reverse transcribed with the use of a TaqMan MiRNA Reverse Transcription Kit (Applied Biosystems) following the manufacturer’s instructions. Of RT products, 2.5 μL was subsequently pre-amplified using TaqMan PreAmp Mastermix and Megaplex PreAmp Primer Pools A + B (Applied Biosystems). The pre-amplification reaction solution was diluted 150-fold with water and combined with equal volume of 2× TaqMan Universal PCR Master Mix (Applied Biosystems). One hundred μL of the sample/master mix was finally loaded into the array, centrifuged and mechanically sealed with the sealer device (Applied Biosystems). Quantitative RT-PCR reactions were performed on the ABI Prism 7900HT Sequence Detection System (Applied Biosystems) according to the cycling conditions recommended by the manufacturer. Acquired data were analyzed with SDS Relative Quantification Software [27]. Serum miRNA expression levels were normalized to cel-miR-238 and the threshold cycle (Ct) values equal to or greater than 40 were defined as undetectable.

### 3.4. Quantification of Candidate miRNAs by Real Time RT-PCR

The expression levels of candidate miRNAs selected from the array analysis were validated with the use of TaqMan miRNA qRT-PCR primers and regents. The amount of 1.67 μL purified RNA from individual serum sample was subjected to RT in a 5 μL reaction mixture using miRNA-specific stem-loop primers and TaqMan miRNA Reverse Transcription Kit (Applied Biosystems) following the manufacturer’s protocol. The real-time PCR assay was carried out in a 10 μL reaction mixture containing 4.5 μL of diluted cDNA (1:15), 5 μL of 2× TaqMan Universal PCR Master Mix (No AmpErase UNG) and 0.5 μL of TaqMan miRNA Assay primers (Applied Biosystems). The thermal cycling conditions were as follows: an initial denaturation step at 95 °C for 10 min, 40 cycles of PCR amplification at 95 °C for 15 s, 60 °C for 1 min. Each serum sample for each candidate miRNA was run in triplicate. The expression level of each miRNA in an individual sample was normalized to cel-miR-238.

### 3.5. Data Analysis

The relative expression level of each miRNA in an individual sample was calculated according to the equation 2^−^^△Ct^, in which △Ct = Ct _miRNA_ − Ct _cel-miR-238_. Then the relative quantification value underwent a Log_2_-transformation to compare the expression levels of candidate miRNAs between healthy controls and patients. Statistical analysis was performed with SPSS software [28] and *p* value *<*0.05 was considered statistically significant. In addition, ROC curve was presented for each miRNA. The AUC values and 95% confidence intervals (CI) were calculated to determine the specificity and sensitivity of H7N9 virus infection. To evaluate the diagnostic potential of serum levels of combined miRNA molecules, multiple logistic regression analysis was conducted as described previously [29].

## 4. Conclusions

The interaction between the virus and the host is a complicated biological process with expression alterations of various molecules involved in development, proliferation, differentiation, apoptosis and immunological regulation. miRNAs, as one of the most important gene expression regulators, have been also implicated in viral infections. Recent studies suggested that host cellular miRNAs played important roles in viral replication and might often be utilized to control viral infection [30,31,32,33,34]. In accordance with these observations, the expression patterns of serum/plasma miRNAs in patients have been found to be significantly changed following various pathogenic infections, although the functions of most of the circulating miRNAs associated with a specific pathological state remain to be elucidated [35,36,37,38,39]. In this study, we observed that nearly 400 miRNA molecules were detected in serum samples of patients with H7N9 virus infection using TaqMan microarray, far beyond the number of miRNAs expressed in healthy individuals. Interestingly, the majority of the commonly expressed 187 serum miRNAs in both groups were up-regulated following this novel virus infection, while only 7 were down-regulated. Although the roles of these cell-free small RNAs during the process of H7N9 virus infection are still unknown, our present findings at least partly reveal the H7N9 virus-host interaction at the miRNA level.

A prominent advantage of circulating miRNAs is their diagnostic and prognostic potential. First, various kinds of body fluid specimens are easily available from individuals. Moreover, serum/plasma miRNAs are highly stable under harsh environments such as extreme PH, boiling and multiple freeze-thaw cycles [40]. These unique characteristics make cell-free small RNAs serve as attractive and powerful markers for the early diagnosis of different diseases, especially cancers such as lung cancer [19], breast cancer [20,21] and colon cancer [41,42]. More recently, the diagnostic capability of cell-free miRNAs in microbial infections were highlighted. A combination of the serum levels of miR-361-5p, miR-889 and miR-576-3p were shown to distinguish pulmonary tuberculosis patients from healthy individuals [26]. Li and colleagues found that four miRNAs, let-7c, miR-23b, miR-122 and miR-150, were differentially expressed in the sera of patients with occult hepatitis virus B infection (OBI) and showed a high level of accuracy in discriminating OBI cases from the non-infected controls [43]. To identify potential biomarkers for this novel H7N9 virus infection, we evaluated four serum miRNAs (miR-17, miR-20a, miR-106a and miR-376c) by ROC curve analysis. It seemed that a combination of these circulating miRNAs could achieve an increased diagnostic efficiency in differentiating H7N9 virus-infected patients from healthy controls compared with each single miRNA.

Host circulating miRNA expression patterns showed significant differences upon different virus infections. HFMD is a viral illness common in children mainly caused by enterovirus infections. A previous study found that 102 were up-regulated and 26 were down-regulated by analyzing the serum miRNA levels in HFMD patients [22]. Further analysis revealed that combined six miRNAs (miR-148a, miR-143, miR-324-3p, miR-628-3p, miR-140-5p and miR-362-3p) could discriminate patients with enterovirus infections from healthy controls. Most recently, Tambyah *et al.* detected the expression profile of miRNAs from blood samples of influenza A H1N1 virus-infected patients and then exhibited that the expression levels of 193 miRNA molecules were altered in all influenza patients, of which 16 highly dysregulated miRNAs (miR-1260, miR-1285, miR-18a, miR-185*, miR-299-5p, miR-26a, miR-30a, miR-335*, miR-34b, miR-519e, miR-576-3p, miR-628-3p, miR-664, miR-665, miR-765 and miR-767-5p) were able to provide a clear distinction between infected and healthy individuals [39]. These differences in cell-free small RNA expression profiles might reflect diverse infectious mechanisms of different viruses. Furthermore, miR-17, miR-20a, miR-106a and miR-376c used in this study for the diagnosis of H7N9 virus infection did not overlap with those for enterovirus and H1N1 virus infections, thus indicating high diagnostic specificity of these four miRNAs. Circulating miR-20a could also serve as a potential predictive biomarker for HCV-mediated fibrosis [23]. Since HCV generally induces chronic infection and patients do not develop respiratory symptoms, H7N9 and HCV viral diseases can be recognized by clinical characteristics independent of circulating miRNA analysis.

Our present study represents a pilot investigation about the responses of host serum miRNAs associated with H7N9 virus infection. It must be noted that much more work needs to be done in the near future to further explore the relation of circulating miRNAs to this emerging reassortant virus. Since the number of research subjects involved in this study is relatively small, a larger number of clinical samples need to be collected to validate our current results. Furthermore, we identified more than 150 serum miRNAs with significant expression differences in patients compared with healthy controls, of which only a very small percentage was evaluated for diagnostic potential. Whether other dysregulated miRNAs could possess higher discriminating efficiency as biomarkers for the diagnosis of H7N9 virus infection might depend on subsequent studies. Finally, an important question remains to be answered: whether the serum levels of four miRNAs (miR-17, miR-20a, miR-106a and miR-376c) could be used for discriminating H7N9 virus from other types of viral infections with the same good performance.

In summary, we, for the first time, provided an overview of host serum miRNA alterations in response to H7N9 virus infection, as well as identified the diagnostic ability of combined miR-17, miR-20a, miR-106a and miR-376c serum levels, thus promoting our understanding of this emerging virus pathogenesis.

## Appendix

**Table A1:** Differential expressed miRNAs in H7N9 virus-infected patients compared with healthy controls.

miRNA	△△Ct	2^−^^△△Ct^
hsa-miR-1290	−10.05	1063.66
hsa-miR-1275	−9.36	655.36
hsa-miR-1260	−9.25	609.09
hsa-miR-574-3p	−4.49	22.48
hsa-miR-454	−4.45	21.93
hsa-miR-148a	−4.38	20.85
hsa-miR-539	−4.36	20.57
hsa-miR-223	−4.27	19.29
hsa-miR-142-5p	−4.27	19.26
hsa-miR-485-3p	−4.21	18.56
hsa-miR-548c-5p	−4.13	17.56
hsa-miR-17	−4.12	17.41
hsa-miR-484	−4.10	17.17
hsa-miR-652	−4.09	17.01
hsa-miR-660	−4.07	16.78
hsa-miR-20b	−4.06	16.66
hsa-miR-511	−4.05	16.61
hsa-miR-26b	−4.01	16.15
hsa-miR-210	−4.01	16.15
hsa-miR-489	−3.98	15.78
hsa-miR-22*	−3.98	15.74
hsa-miR-15a*	−3.97	15.70
hsa-miR-106a	−3.92	15.15
hsa-miR-331-5p	−3.91	15.04
hsa-miR-194	−3.88	14.73
hsa-miR-139-5p	−3.85	14.38
hsa-miR-193a-5p	−3.84	14.37
hsa-miR-29a	−3.80	13.94
hsa-miR-24	−3.75	13.43
hsa-miR-140-5p	−3.71	13.12
hsa-miR-28-3p	−3.69	12.91
hsa-miR-151-3p	−3.67	12.73
hsa-miR-192	−3.66	12.62
hsa-miR-190b	−3.60	12.15
hsa-miR-143	−3.59	12.04
hsa-miR-425	−3.58	11.96
hsa-miR-101	−3.55	11.70
hsa-miR-146b-5p	−3.52	11.45
hsa-miR-26a	−3.47	11.06
hsa-miR-425*	−3.46	11.02
hsa-miR-25	−3.45	10.92
hsa-miR-186	−3.44	10.86
hsa-miR-191	−3.42	10.72
hsa-miR-9*	−3.42	10.72
hsa-miR-494	−3.41	10.65
hsa-miR-323-3p	−3.41	10.64
hsa-miR-34a	−3.27	9.65
hsa-miR-125a-5p	−3.26	9.58
hsa-miR-152	−3.25	9.51
hsa-miR-19b	−3.24	9.45
hsa-miR-532-5p	−3.23	9.41
hsa-miR-324-5p	−3.21	9.25
hsa-miR-106b	−3.19	9.12
hsa-miR-886-5p	−3.19	9.11
hsa-miR-744	−3.14	8.83
hsa-miR-451	−3.13	8.75
hsa-miR-130a	−3.13	8.73
hsa-miR-200c	−3.11	8.63
hsa-miR-625*	−3.11	8.61
hsa-miR-532-3p	−3.10	8.56
hsa-miR-720	−3.08	8.44
hsa-miR-338-5p	−3.05	8.30
hsa-miR-324-3p	−3.05	8.29
hsa-miR-203	−3.05	8.27
hsa-miR-30a-3p	−3.01	8.07
hsa-miR-345	−3.01	8.04
hsa-miR-27a	−2.92	7.57
hsa-miR-30e-3p	−2.88	7.37
hsa-miR-664	−2.86	7.28
hsa-miR-340	−2.77	6.84
hsa-miR-495	−2.76	6.79
hsa-miR-21	−2.75	6.74
hsa-miR-335	−2.72	6.58
hsa-miR-181a	−2.71	6.53
hsa-miR-218	−2.62	6.16
hsa-miR-223*	−2.58	5.99
hsa-miR-93*	−2.57	5.95
hsa-miR-151-5p	−2.52	5.74
hsa-miR-27b	−2.51	5.70
hsa-miR-199a-5p	−2.47	5.56
hsa-miR-505	−2.47	5.53
hsa-miR-93	−2.38	5.21
hsa-miR-144*	−2.34	5.06
hsa-let-7g	−2.34	5.06
hsa-miR-130b	−2.32	5.00
hsa-miR-128	−2.23	4.70
hsa-miR-132	−2.22	4.66
hsa-miR-331-3p	−2.10	4.29
hsa-miR-375	−2.07	4.20
hsa-miR-125b	−2.02	4.06
hsa-miR-148b	−2.00	4.00
hsa-miR-19b-1*	−2.00	3.99
hsa-miR-122	−1.90	3.73
hsa-miR-486-5p	−1.86	3.64
hsa-miR-409-3p	−1.85	3.60
hsa-miR-142-3p	−1.81	3.50
hsa-miR-150	−1.80	3.48
hsa-let-7b	−1.76	3.38
hsa-miR-541*	−1.73	3.32
hsa-miR-221	−1.62	3.08
hsa-miR-649	−1.61	3.06
hsa-miR-92a	−1.54	2.92
hsa-miR-518f	−1.33	2.51
has-miR-155	−1.28	2.43
hsa-miR-301a	−1.12	2.17
hsa-miR-486-3p	−1.07	2.10
hsa-miR-10a	−1.05	2.07
hsa-miR-642	1.09	0.47
hsa-miR-654-3p	1.22	0.43
hsa-let-7e	1.28	0.41
hsa-miR-548c-3p	1.34	0.39
hsa-miR-29b	1.41	0.38
hsa-miR-548p	1.77	0.29
hsa-miR-1243	9.65	0.00

The different Ct value between two groups was calculated by △△Ct method. The ratio of miRNAs in two groups was calculated by using the equation 2*^−^*^ΔΔCT^.
